# New Appliances and Things Medical

**Published:** 1906-07-07

**Authors:** 


### NEW APPLIANCES AND THINGS MEDICAL.

[We shall be glad to receive, at our Office, 28 & 29 Southampton Street, Strand, London, W.C., from the manufacturers, specimens of all new preparations and appliances which may be brought out from time to time.]

### ADLEY, TOLKEIN AND CO.'S PREPARATIONS.

(Manufacturing Chemists, Blackburn, England.)

We have received from this firm samples of three important preparations. The first that we shall consider is the creosoted emulsion of cod-liver oil with hypophosphites. Each fluid ounce of this preparation contains two drachms of cod-liver oil, five grains of Beechwood creosote, two grains of sodium hypophosphite, and three grains of calcium hypophosphite. The ordinary dose is one teaspoonful three times a day. The value of the above ingredients, both separately and together, in certain pathological states is well established, and this preparation is exceedingly palatable and reliable.

The second product of this firm is an ethereal antiseptic soap. This soap contains, as an antiseptic, oil of wintergreen (methyl-salicylate); it forms an excellent lather, and by virtue of its alcohol and ether content is very potent in removing grease from the skin, thus allowing intimate contact to take place between the latter and the antiseptic.

The third preparation submitted to us is called Poulticine. This substance is a substitute for poultices, rubefacients, and counter-irritants generally. It is supplied in tins, and must be melted by allowing the tin to stand in hot water for 15 minutes. The viscid liquid is then applied to the required area in a layer of about $\frac{1}{8}$ of an inch thick; this is subsequently covered with cotton-wool or lint, which latter is held by a flannel bandage. An effect identical to that of a poultice ensues.

